# Blockade of C5aR1 alleviates liver inflammation and fibrosis in a mouse model of NASH by regulating TLR4 signaling and macrophage polarization

**DOI:** 10.1007/s00535-023-02002-w

**Published:** 2023-05-25

**Authors:** Keqing Jiang, Shibang Lu, Dongxiao Li, Mingjiang Liu, Hu Jin, Biao Lei, Sifan Wang, Kang Long, Songqing He, Fudi Zhong

**Affiliations:** 1 Division of Hepatobiliary Surgery, The First Affiliated Hospital of Guangxi Medical University, Nanning, 530021 China; 2 Guangxi Key Laboratory of Immunology and Metabolism for Liver Diseases, Guangxi Medical University, Nanning, 530021 China; 3 Key Laboratory of Early Prevention and Treatment for Regional High Frequency Tumor (Guangxi Medical University), Ministry of Education, Nanning, 530021 China

**Keywords:** Nonalcoholic steatohepatitis, Complement factor C5a, C5aR1, Toll-like receptor signaling pathway, Macrophage polarization

## Abstract

**Background:**

Nonalcoholic steatohepatitis (NASH) is an advanced form of chronic fatty liver disease, which is a driver of hepatocellular carcinoma. However, the roles of the C5aR1 in the NASH remain poorly understood. Here, we aimed to investigate the functions and mechanisms of the C5aR1 on hepatic inflammation and fibrosis in murine NASH model.

**Methods:**

Mice were fed a normal chow diet with corn oil (ND + Oil), a Western diet with corn oil (WD + Oil) or a Western diet with carbon tetrachloride (WD + CCl_4_) for 12 weeks. The effects of the C5a–C5aR1 axis on the progression of NASH were analyzed and the underlying mechanisms were explored.

**Results:**

Complement factor C5a was elevated in NASH mice. C5 deficiency reduced hepatic lipid droplet accumulation in the NASH mice. The hepatic expression levels of TNFα, IL-1β and F4/80 were decreased in C5-deficient mice. C5 loss alleviated hepatic fibrosis and downregulated the expression levels of α-SMA and TGFβ1. C5aR1 deletion reduced inflammation and fibrosis in NASH mice. Transcriptional profiling of liver tissues and KEGG pathway analysis revealed that several pathways such as Toll-like receptor signaling, NFκB signaling, TNF signaling, and NOD-like receptor signaling pathway were enriched between C5aR1 deficiency and wild-type mice. Mechanistically, C5aR1 deletion decreased the expression of TLR4 and NLRP3, subsequently regulating macrophage polarization. Moreover, C5aR1 antagonist PMX-53 treatment mitigated the progression of NASH in mice.

**Conclusions:**

Blockade of the C5a–C5aR1 axis reduces hepatic steatosis, inflammation, and fibrosis in NASH mice. Our data suggest that C5aR1 may be a potential target for drug development and therapeutic intervention of NASH.

**Supplementary Information:**

The online version contains supplementary material available at 10.1007/s00535-023-02002-w.

## Introduction

Nonalcoholic steatohepatitis (NASH) is characterized by the presence of hepatocyte ballooning, necroinflammation, and fibrosis [1]. NASH has emerged as the major cause of cryptogenic cirrhosis and even hepatocellular carcinoma (HCC) worldwide. Owing to the high prevalence of nonalcoholic fatty liver disease (NAFLD), NASH is the third leading indication for liver transplantation in the USA [2, 3]. Some studies advised patients to change their lifestyle, including diet and exercise therapies, to gain weight loss in the management of NAFLD [4–6], but the approved therapies for NASH are still limited. Currently, several drug trials for NAFLD/NASH therapy are ongoing [5, 6]; however, there are no FDA-approved drugs for treatment of NASH.

The etiology of NASH is complex and causation is multifactorial. The primary insult of lipid accumulation is followed by various pathogenic drivers, such as oxidative stress [7], endoplasmic reticulum (ER) stress [8], and innate immune system activation. Innate immune activation is the key factor which triggers and amplifies liver inflammation, promoting the development of NASH [9]. The complement system is a major arm of innate immunity and plays an important role in the pathogenesis of NAFLD [10]. Rensen et al. [11] observed that complement C3 and mannose binding lectin were deposited around hepatocytes with lipid accumulation and neutrophil infiltration in NAFLD patients. Another study had shown that the level of serum complement C3 is positively correlated with the prevalence and severity of NAFLD [12]. Moreover, the serum level of C5a, one component of C3 downstream, is increased in obese children and positively correlated with body mass index, waist circumference, triglycerides (TG), and insulin resistance [13]. Animal experimental studies showed that murine complement C5 contributes to nonalcoholic liver steatosis and the progression of inflammation [14, 15]. A previous study indicated that complement C5a receptor (C5aR) is closely associated with inflammation in obese adipose tissue [10]. These results suggest that the activation of the complement system is involved in the pathogenesis of NAFLD.

Complement C5 was identified as a critical factor for liver fibrosis in mice and humans [16]. It has been reported that the increase in C5a concentration is positively correlated with the severity of liver fibrosis in patients with chronic hepatitis B [17]. Another study showed that C5 deficiency can delay the progression of biliary fibrosis in bile duct-ligated mice [18]. A previous study demonstrated that C5aR1 has a pathogenic role in chronic inflammation and renal fibrosis in a murine model of chronic pyelonephritis [19]. Peng et al. [20] reported that C5a and C5aR1 interaction promotes progression of renal tubulointerstitial fibrosis in ischemia/reperfusion injury. However, the effect of the C5a–C5aR1 axis on the fibrosis in NASH remains largely unknown.

It is difficult to induce fibrosis and severe NASH in mice fed with Western diet, even with long-term feeding for 25 weeks or longer [21]. To test the function of C5a–C5aR1 on the fibrosis in NASH, we used a Western diet with low-dose CCl_4_-induced NASH model with rapid progression of fibrosis and severe NASH [22]. In this study, we further explored the effect of C5a–C5aR1 axis on hepatic steatosis, inflammation, and fibrosis in a NASH model and its underlying mechanisms.

## Materials and methods

### Mice and NASH model

C5^−/−^ (B10.D2-*Hc*^*0*^*H2*^*d*^*H2-T18*^*c*^/0SnJ) mice and their haplotype (B10.D2-*Hc*^*1*^*H2*^*d*^*H2-T18*^*c*^/nSnJ) were purchased from the Jackson Laboratory (Bar Harbor, ME, USA). C5aR1^−/−^ mice with a C57BL/6 background were purchased from GemPharmatech (Jiangsu, China). All mice were maintained in a specific pathogen-free facility at Guangxi Medical University. A Western diet (WD) and chemically induced murine NASH model as described previously [22] were used. Briefly, male mice were fed normal chow diet/corn oil (ND + Oil), WD/corn oil (WD + Oil) or WD combined with low weekly dose of intraperitoneal carbon tetrachloride (CCl_4_) for 12 weeks. Male C5^−/−^ and their haplotype, C5aR1^−/−^ mice and C57BL/6 wild-type mice, aged 8–12 weeks, were fed a WD/CCl_4_ (WD + CCl_4_) for 12 weeks. Corn oil or CCl_4_ were intraperitoneally injected into male mice once a week. All animal experiments were approved by the Animal Care and Use Committee of Guangxi Medical University, Guangxi, China.

### Histopathologic and immunohistochemistry (IHC)

Fresh liver tissues were frozen, and sliced into 8 μm thick sections. The sections were stained with an Oil Red O staining. Liver biopsy specimens were also fixed with 10% neutral formalin and embedded in paraffin, and then tissue sections (5 μm thick) were stained with hematoxylin–eosin (H&E) and Sirius Red. Histopathological changes in the liver biopsies were assessed using a NanoZoomer S60 digital slide scanner (Hamamatsu, Japan).

Paraffin-embedded sections (4 μm thick) were processed for immune-histochemical staining. Antigen retrieval was performed by pressure cooking for 5 min in citrate buffer (pH 6), followed by peroxidase and serum blocking steps. The sections were incubated with goat anti-C3d antibody (R&D Systems, 1:100 dilution), anti-F4/80 (CST, 1:500 dilution) or anti-α-SMA (Abcam, 1:500 dilution) for 2 h at room temperature, followed by antibody detection with an anti-goat ImmPRESS kit (Vector Laboratories). The images were collected using the NanoZoomer S60 digital slide scanner.

### Biochemical indicators and serum C5a analyses

Following killing, retro-orbital blood of the experimental mice was collected under isoflurane anesthesia to obtain serum for analysis. Serum alanine aminotransferase (ALT) and aspartate aminotransferase (AST) levels were measured with an autoanalyzer (ANTECH Diagnostics, Los Angeles, CA, USA). Serum C5a level was measured using commercially available ELISA kits (CUSABIO, Wuhan, China), according to the manufacturer’s instructions.

### Measurement of triglyceride (TG), malondialdehyde (MDA), and glutathione (GSH) levels in liver homogenates

The levels of TG, MDA, and GSH in liver tissue homogenates from each group were measured using the corresponding kits (Catalog# A110-1, A003-1 and A006-2, respectively) from Nanjing Jiancheng Bioengineering Institute (Nanjing, China) according to the manufacturer’s protocols.

### Western blot analysis

Proteins of hepatic samples or cells were analyzed using standard western blotting techniques. The antibodies of anti-α-SMA (ab32575, Abcam), anti-TGF-β1 (SAB4502954, Sigma), anti-P65 (10,745–1-AP, Proteintech), anti-p-P65 (AF2006, Affinity), anti-iNOS (13120S, CST), anti-CD86 (19,589, CST), anti-CD163 (16,646–1-AP, Proteintech), anti-CD206 (24,595, CST), anti-TLR4 (19,811–1-AP, Proteintech), anti-NLRP3 (A5652, ABclonal), anti-AKT (10,176–2-AP, Proteintech), anti-p-AKT (4060S, CST), anti-HO-1 (43,966, CST), anti-SOD1 (10,269–1-AP, Proteintecch), anti-MDA (MDA11-S, ADI), or anti-CASP1 (24232S, CST) were used as primary antibodies. Anti-GAPDH (10,494–1-AP, Proteintech) or anti-TUBULIN (#2144, CST) was used to normalize the signals. Bands were quantified by ImageJ software.

### RNA isolation and quantitative (q) RT-PCR

Total RNA was extracted from 0.1 g frozen hepatic tissues according to the TRIzol reagent protocol (No. 15596–026, Invitrogen). Next, 2 μg total RNA was reverse transcribed to complementary DNA (cDNA) using the RevertAid First Strand cDNA Synthesis Kit (No.K1622, Thermo Scientific) according to the manufacturer’s protocol. Relative mRNA levels of genes were measured by qRT-PCR, using a SYBR Green PCR master mix (No.1725125, Bio-Rad). All experiments were performed in triplicate. The qRT-PCR primers used in this study were shown in supplementary Table 1. GAPDH was used to normalize the signals of target gene in the same sample.

### Transcriptional profiling

Total RNA was extracted from flash-frozen liver tissues of *C5aR1*^*−/−*^ and WT NASH mice with TRIzol reagent (Invitrogen, Carlsbad, CA, USA). The quality of the RNA samples was evaluated with a NanoDrop 2000c spectrophotometer (Thermo Fisher Scientific, Waltham, MA, USA) and an Agilent’s Bioanalyzer. Sequencing libraries were generated by reverse transcription-polymerase chain reaction (RT-PCR) amplification. The PCR products were sequenced on a HiSeq 2500 sequencing system (RIBOBIO, Guangzhou, China). Transcriptional profiling data were deposited at Mendeley Data (https://data.mendeley.com), which can easily be accessed at http://dx.doi.org/10.17632/j8v2c8z7zt.1

### Cell experiments

RAW264.7 cells were kindly provided by Kunming Cell Bank of Typical Culture Preservation Committee of Chinese Academy of Sciences, Kunming, China. RAW264.7 cells were cultured in Dulbecco’s modified Eagle medium (C11995500BT, Gibico) supplemented with 10% fetal bovine serum. The cells were grown at 37 °C in an atmosphere of 5% CO_2_. The RAW264.7 cells were cultured overnight to about 80% confluent and treated by the recombinant protein C5a (HY-P7695, MCE) with 50 ng/mL for 24 h.

### Antagonist PMX-53 treatment

The antagonist PMX-53 was purchased from Nanjing Peptide Industry (Nanjing, China) and diluted in saline. PMX-53 (1 mg/kg) was administered i.p. to the experimental group and saline administration served as the control for the last 4 weeks of the experimental protocol. Mice subject to NASH model were treated with PMX-53 every other day from week eight forward.

### Data analysis

Data are shown as the mean ± standard deviation. Significant differences between groups were determined by ANOVA, with a Bonferroni correction for continuous variables and multiple groups. Two-tailed Student’s *t* test was used for the comparison of a normally distributed continuous variable between two groups. Nonparametric statistical analysis was performed using the Mann–Whitney test. Statistical analysis was performed using GraphPad Prism (Version 7.0.4) software. *P* values less than 0.05 were considered as statistically significant.

## Results

### Complement activation in chemically induced NASH mice

**Fig. 1:**
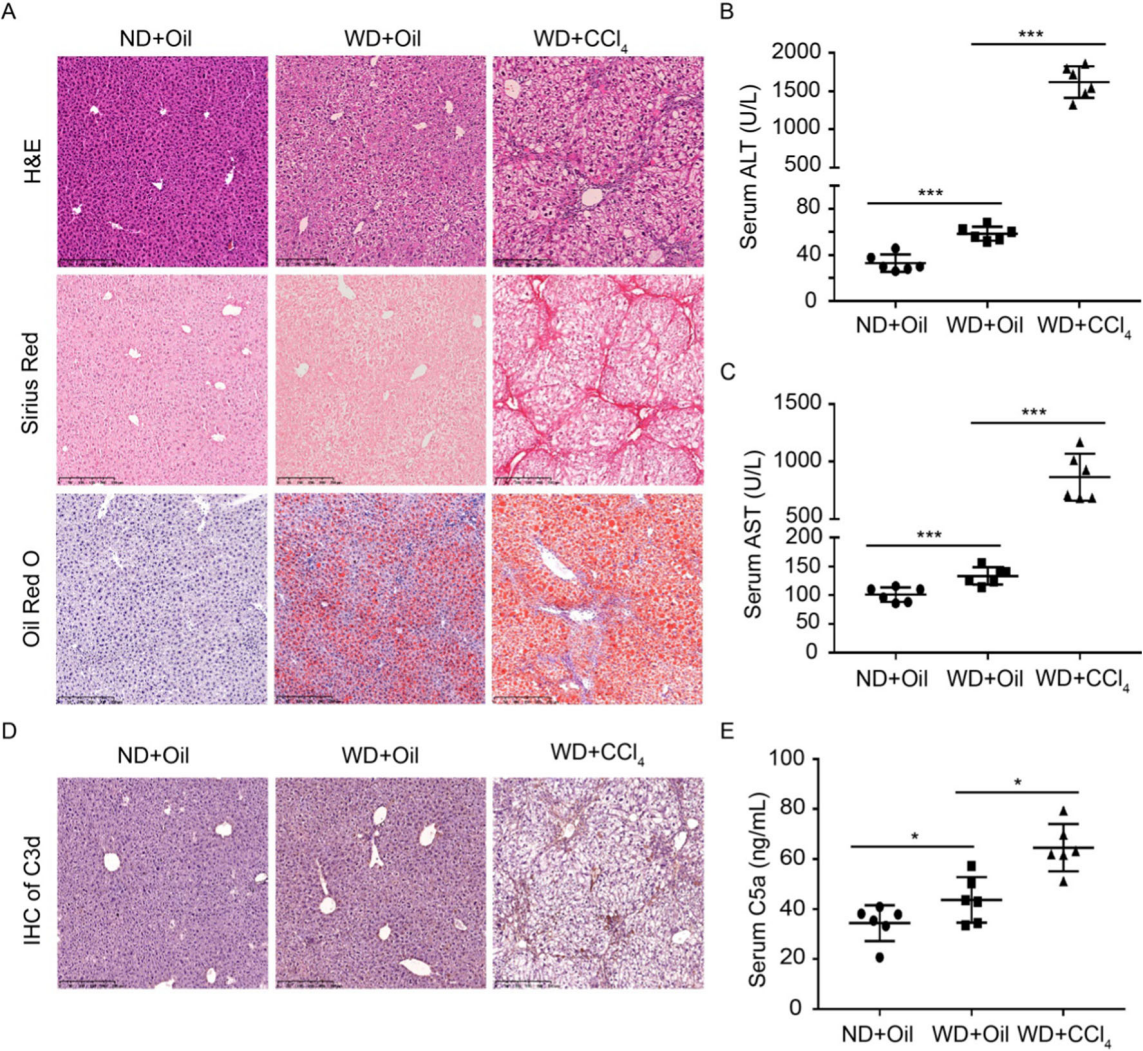
Complement levels and liver injury are elevated in NASH mice. **A** Liver tissue sections were assayed by H&E staining (upper), Sirius Red staining (mid), and Oil Red O staining (lower). Scale bar: 250 μm. **B** Serum level of ALT. **C** Serum level of AST. **D** Immunohistochemical staining of C3d. Scale bar: 250 μm. **E** Serum level of C5a was measured by ELISA. Results are presented as mean ± SD. *P < 0.05, ***P < 0.001. *n* = 6 per group

To investigate whether the complement system was activated in NASH, mice were subjected to a normal diet/corn oil (ND + Oil), Western diet/corn oil (WD + Oil) and WD combined with a low weekly dose of intraperitoneal CCl_4_ (WD + CCl_4_) as described previously [22], resulting in a rapid progression to NASH with fibrosis. The results of H&E and Sirius Red staining showed that hepatic fibrosis was not induced in mice treated with WD + Oil, but increased in mice treated with WD + CCl_4_ compared to ND + Oil group (Fig. 1A). Oil Red O staining indicated that hepatic lipid droplets were accumulated in the groups of WD + Oil and WD + CCl_4_ compared with ND + Oil group (Fig. 1A). Serum levels of alanine aminotransferase (ALT) and aspartate transaminase (AST) were slightly increased in the WD + Oil group relative to ND + Oil, and significantly raised in WD + CCl_4_ group compared to ND + Oil and WD + Oil (Fig. 1B, C). Immunohistochemical staining revealed that extensive C3d was deposited in the WD + Oil and WD + CCl_4_ group (Fig. 1D). Furthermore, the serum level of C5a was elevated in the WD + Oil compared with ND + Oil, and obviously increased in the WD + CCl_4_ group compared with ND + Oil and WD + Oil groups (Fig. 1E). Taken together, our results demonstrate that complement level and liver injury are significantly elevated in the NASH mice.

### C5 deficiency alleviated the hepatic steatosis and inflammation in NASH mice

**Fig. 2:**
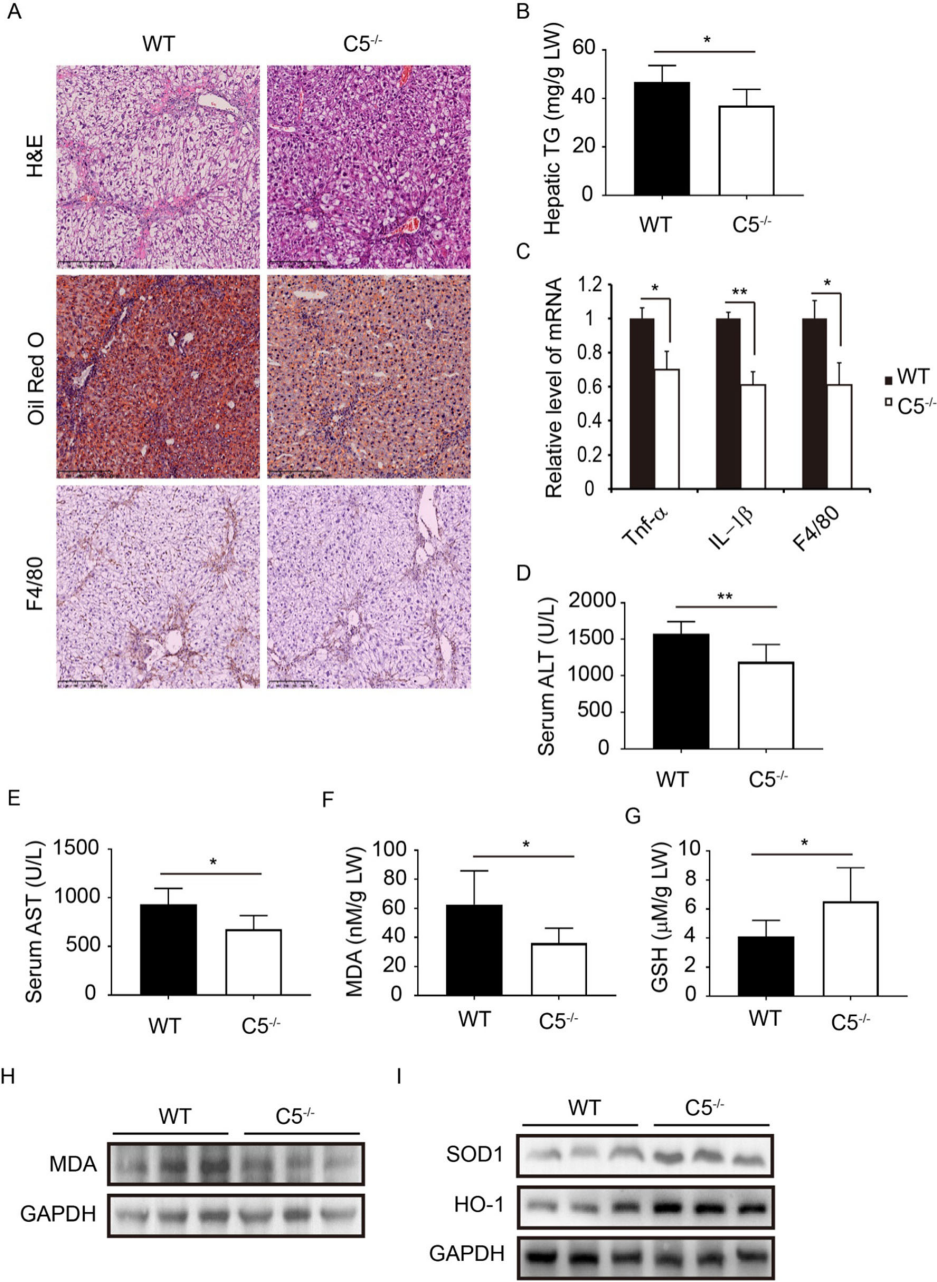
C5 deficiency attenuates hepatic steatosis and inflammation in NASH mice. **A** Liver tissue sections were assessed by H&E (upper), Oil Red O staining (mid), or Immunohistochemistry (IHC) of F4/80 (lower). Scale bar: 250 μm. **B** The level of triglyceride in liver homogenate. **C** Relative mRNA levels of Tnf-α, IL-1β and F4/80 in liver were examined by qRT-PCR. The data is representative of three independent experiments. **D** Serum level of ALT. **E** Serum level of AST. **F** The MDA content of liver homogenate. **G** The GSH level in liver tissues. *LW* liver weight. Results are presented as mean ± SD. **H** The protein level of MDA was measured by western blot. **I** The protein levels of SOD1 and HO-1 were examined by western blot. *P < 0.05, **P < 0.01.* n* = 6 per group

Previous studies had shown that C5 is correlated to the progression of NAFLD [14, 15]. Thus, we sought to investigate the effects of C5 on the hepatic steatosis and inflammation in the NASH model. Compared to WT mice, the results of H&E and Oil Red O staining revealed that C5 deficiency reduced hepatic lipid droplet accumulation in the NASH mice (Fig. 2A). In addition, the level of triglyceride (TG) in liver tissue was decreased in the C5 deficient mice (Fig. 2B). The results of the qRT-PCR analysis showed that the mRNA levels of inflammation associated genes such as Tnf-α, IL-1β and F4/80 were downregulated in the liver of C5 deficient mice (Fig. 2C). Immunohistochemical analysis showed that hepatic F4/80 deposition was reduced in C5 deficient mice compared to WT mice (Fig. 2A). Moreover, serum levels of ALT and AST were decreased in the C5 deficient mice compared with WT mice (Fig. 2D and E). As we know, hepatic lipid accumulation leads to oxidative stress, which is correlated to the progression of inflammation. Therefore, we further examined the effect of C5 absence on the expression of oxidative stress markers. The liver homogenate level of MDA was decreased in the C5 deficient mice; in contrast, the level of GSH was increased (Fig. 2F and G). Moreover, western blot examination showed that the protein level of MDA was downregulated, in contrast, the protein levels of heme oxygenase 1 (HO-1) and superoxide dismutase 1 (SOD1) were upregulated in the liver of C5^−/−^ mice compared with WT mice (Fig. 2H and I). These results indicate that C5 deficiency decreases the oxidative stress and ameliorates hepatic steatosis and inflammation in NASH mice.

### C5 deficiency suppresses the development of liver fibrosis in chemically induced NASH

**Fig. 3:**
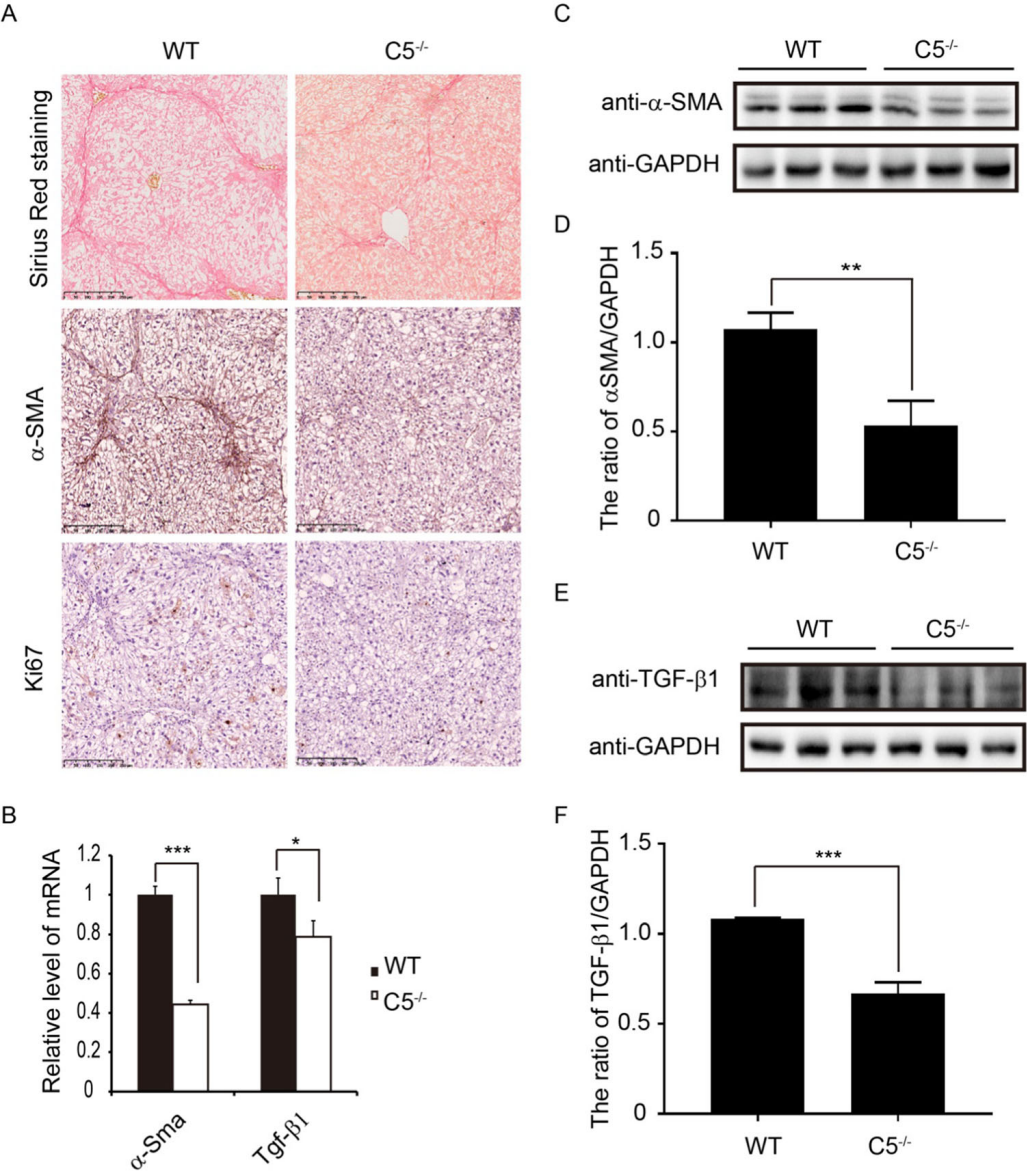
C5 deficiency alleviates liver fibrosis in the NASH mice. C5^−/−^ and WT mice were treated with WD + CCl_4_. **A** Liver tissue sections were assessed by Sirius Red staining (upper), immunohistochemical staining of α-SMA (mid) or Ki67 (lower). Scale bar: 250 μm. **B** The mRNA levels of hepatic α-Sma and Tgf-β1 were analyzed by qRT-PCR. The data is representative of three independent experiments. **C** The protein level of α-SMA was assayed by western blot. **D** Quantitative analysis of the ratio of α-SMA/GAPDH. **E** The protein level of TGF-β1 was examined by western blot. **F** Quantitative analysis of the ratio of TGF-β1/GAPDH. The experiment was repeated three times. Results are presented as mean ± SD. *P < 0.05, **P < 0.01, ***P < 0.001

A previous study reported that C5 has a causal role in liver fibrogenesis [16], but its role in the hepatic fibrosis of chemically induced NASH remains unclear. To explore the effects of C5 on the hepatic fibrosis in the NASH model, wild-type (WT) and C5^−/−^ mice were fed WD and injected with low-dose CCl_4_ for 12 weeks. Then, analysis of hepatic fibrosis was performed by Sirius Red staining and immunohistochemical staining of α-smooth muscle actin (α-SMA). We found that compared to the WT mice, C5 deficiency resulted in a reduction of liver fibrosis in the NASH model (Fig. 3A). Immunohistochemical staining analysis showed that C5 deficient mice had decreased staining of α-SMA compared to WT (Fig. 3A). In addition, staining of Ki67 showed that loss of C5 inhibited cell proliferation (Fig. 3A). Again, C5 deficiency diminished the expression of several pro-fibrotic associated genes, such as α-Sma and Tgf-β1 (Fig. 3B). We further examined the protein level of α-SMA, a marker of activated hepatic stellate cells (HSCs), and TGF-β1 in the liver tissue using western blot. The protein levels of α-SMA and TGF-β1 were clearly decreased in the C5 deficient mice (Fig. 3C–F). These data demonstrate that C5 deficiency protects against the progression of liver fibrosis in NASH mice.

### C5aR1 deficiency slows the progression of NASH

**Fig. 4:**
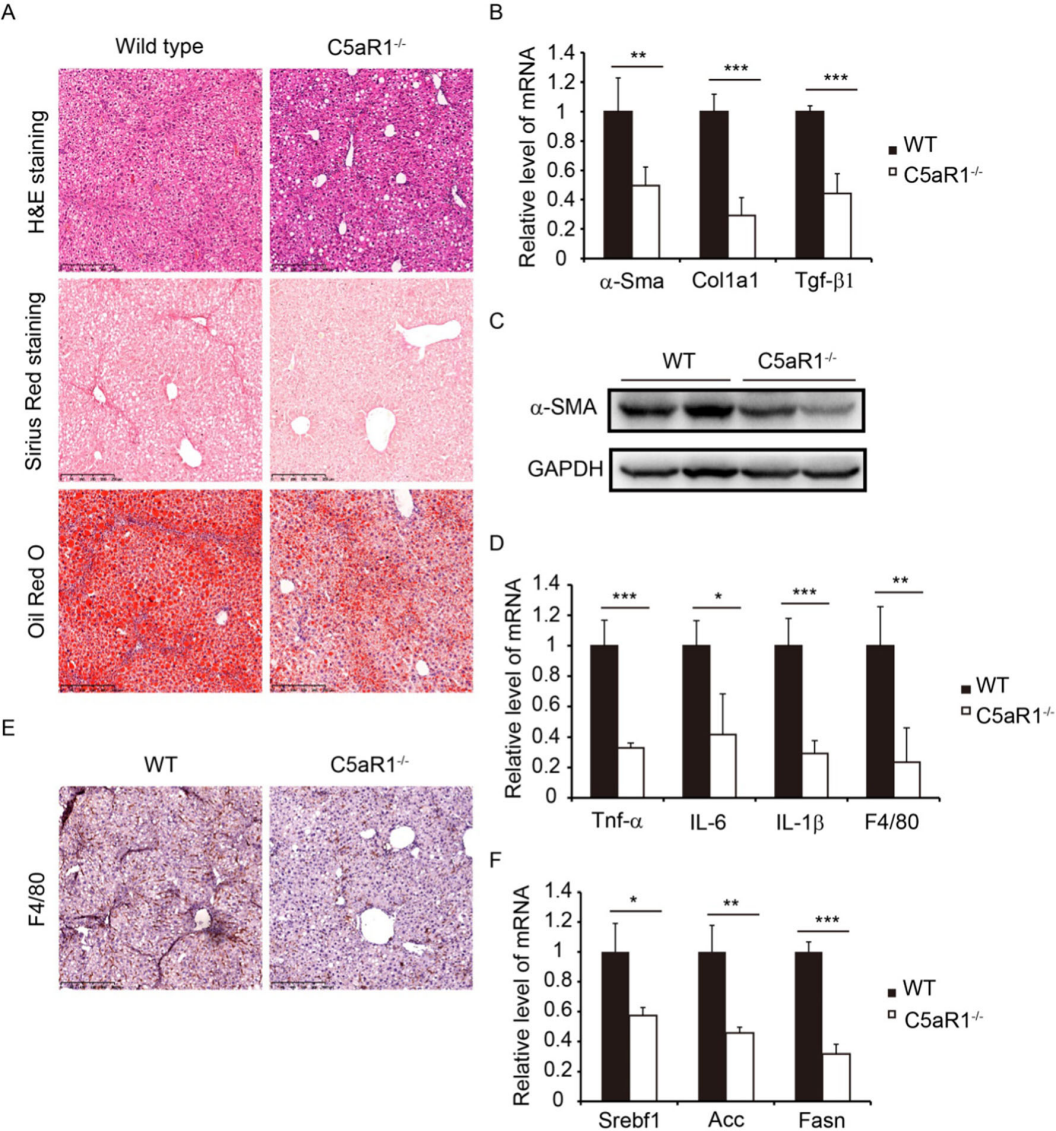
C5aR1 deletion ameliorated the liver inflammation and fibrosis in NASH mice. **A** Liver tissue sections were assessed by H&E staining (upper), Sirius Red staining (mid), and Oil Red O staining (lower). Scale bar: 250 μm. **B** The mRNA levels of α-Sma, Col1a1, and Tgf-β1. **C** The protein level of α-SMA was examined by western blot. **D** The mRNA levels of Tnf-α, IL-6, IL-1β, and F4/80. **E** Immunohistochemical staining of F4/80. Scale bar: 250 μm. **F** The mRNA levels of Srebf1, Acc, and Fasn were determined by qRT-PCR. The data is representative of three independent experiments. *P < 0.05, **P < 0.01, ***P < 0.001. *n* = 6 per group

While C5a/C5aR1 interactions have been identified as an important driver of inflammation and fibrosis, their function in NASH progression in mice has not been completely elucidated. Therefore, C5aR1^−/−^ mice were maintained on the NASH model of WD + CCl_4_ to functionally validate the effect of C5aR1 on the development of NASH. Histopathology and Sirius Red staining revealed that C5aR1 deficiency alleviated the fibrosis level in NASH mice (Fig. 4A). Moreover, the expression of pro-fibrotic markers, such as α-Sma, Col1a1, and Tgf-β1 was diminished in the liver tissue of C5aR1 deleted mice (Fig. 4B). The decreased protein level of α-SMA was further confirmed by western blot (Fig. 4C). Again, the mRNA levels of inflammation associated genes Tnf-α, IL-6 and IL-1β were downregulated in C5aR1^−/−^ mice compared with WT mice (Fig. 4D). Immunohistochemical staining showed that the deposition of F4/80 was clearly reduced in the liver of C5aR1 deficient mice (Fig. 4E), which is consistent with the qRT-PCR result of F4/80 (Fig. 4D). In addition, Oil Red O staining analysis showed that hepatic lipid droplets were reduced in C5aR1^−/−^ mice compared to WT mice (Fig. 4A). C5aR1 deletion markedly decreased the expression of lipogenesis genes such as Srebf1, Acc and Fasn in liver (Fig. 4F). Taken together, we conclude that loss of C5aR1 inhibits the progression of NASH, including resistance to steatosis, inflammation, and fibrosis.

### Mechanism of C5aR1 deficiency in preventing the progression of NASH in mice

**Fig. 5:**
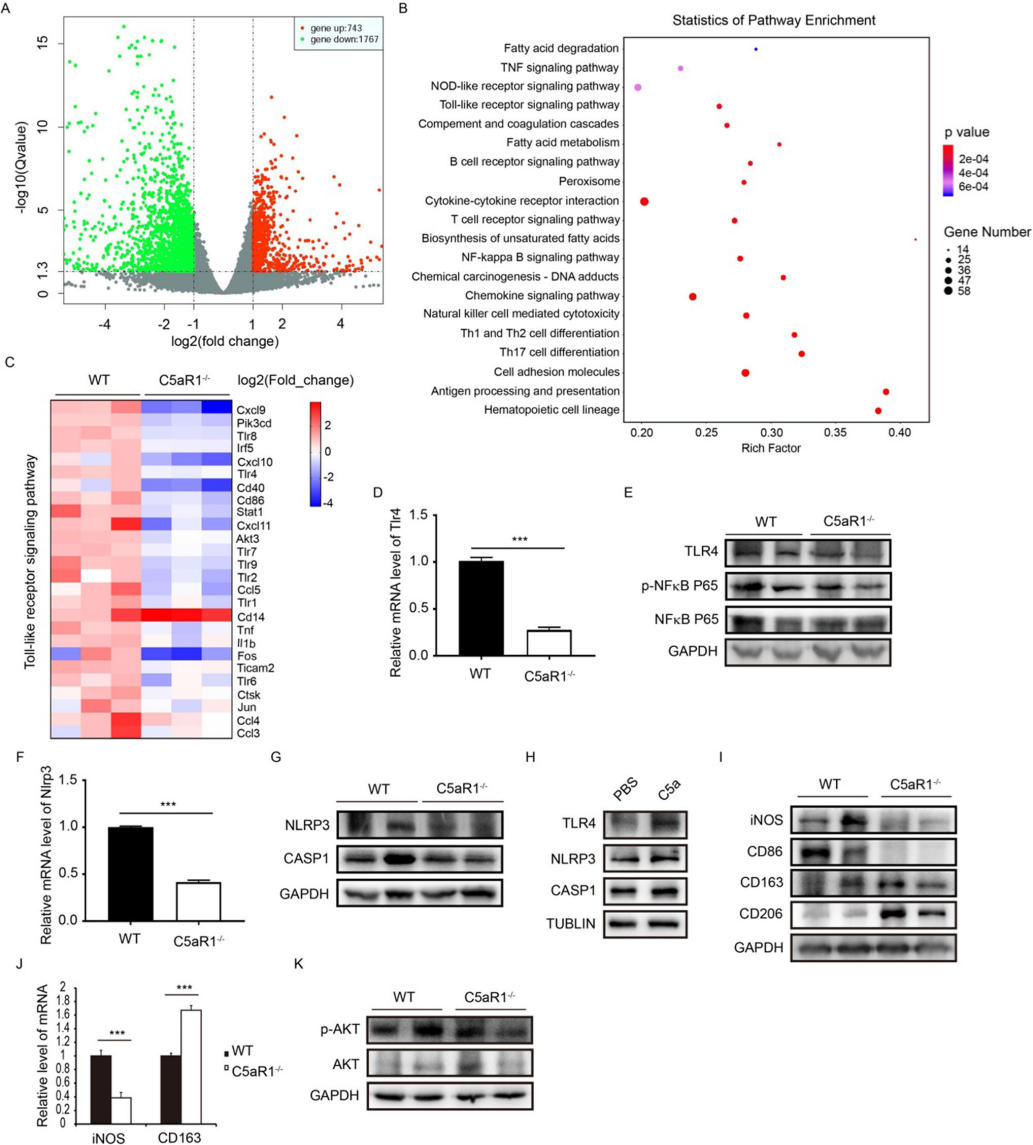
Transcriptional profiling of the liver in NASH mice and verification of NASH associated signaling pathways. **A** Transcriptional profiling of the liver tissue of C5aR1^−/−^ and WT mice. **B** KEGG pathway analysis. **C** Toll-like receptor signaling pathway associated genes were showed in heatmap. **D** The mRNA level of Tlr4 was measured by qRT-PCR. The data is representative of three independent experiments. **E** The protein levels of TLR4, p-NFκB P65 and NFκB P65. **F** The mRNA level of Nlrp3 was determined by qRT-PCR. **G** The protein levels of NLRP3 and CASP1. **H** RAW264.7 cells were treated with the recombinant protein C5a (50 ng/mL) for 24 h. The protein levels of TLR4, NLRP3, and CASP1 were determined by western blot. **I** The protein levels of iNOS, CD86, CD163 and CD206 were measured by Western blot. **J** The mRNA levels of iNOS and CD163. K. Protein levels of p-AKT and AKT. Experiments were repeated three times. Results are presented as mean ± SD. ***P < 0.001. *n* = 6 per group

To explore the putative mechanisms of C5aR1 in NASH, transcriptional profiling of liver tissues from NASH mice was performed. There were more than 2500 differentially expressed genes in the liver tissues of C5aR1^−/−^ and WT mice (Fig. 5A). KEGG pathway analysis revealed that several pathways such as Toll-like receptor signaling pathway, NFκB signaling pathway, TNF signaling pathway, and NOD-like receptor signaling pathway associated with NASH was enriched between C5aR1^−/−^ and WT mice (Fig. 5B). We hypothesized that C5aR1 deficiency slowed the development of NASH by regulating these signaling pathways. Toll-like receptor signaling pathway associated genes were further analyzed and we found that some genes such as TLR4, CD86, and Tnf were significantly decreased in C5aR1 deleted mice (Fig. 5C). Firstly, we verified the expression of some key genes related to these pathways. The result of qRT-PCR showed that the mRNA level of TLR4 was decreased in C5aR1 deficient NASH mice (Fig. 5D), but not affected in mice fed a normal diet (Supplementary Fig. 1). Western blot confirmed that the protein level of TLR4 was downregulated in the C5aR1^−/−^ mice compared to WT mice (Fig. 5E). As we know, Toll-like receptor signaling pathway regulates the downstream pathway NFκB signaling pathway. Therefore, the expression levels of some key genes associated with NFκB pathway were also assessed by western blot. As expected, western blot analysis showed that the ratio of p-NFκB P65/ NFκB P65 was decreased in the C5aR1 deficient mice (Fig. 5E). NFκB signaling pathway, which mediates the expression of TNFα and IL-6, is a crucial mediator of inflammation. Our above data showed that C5aR1 deletion exactly reduced the expression of TNFα and IL-6 (Fig. 4D). Previous studies showed that TLR4 signaling is involved in mediating inflammasome activation [23, 24]. Therefore, the effect of C5aR1 on the NLRP3 signaling was assessed. Our result showed that the mRNA and protein level of NLRP3 were reduced in C5aR1 deficient mice (Fig. 5F and G). The downstream factors of NLRP3 signaling, CASP1 and IL-1β, were decreased by the C5aR1 deletion (Fig. 5G, and Fig. 4D). In addition, RAW264.7 cells were treated with the recombinant protein C5a and we found that C5a treatment significantly increased the expression of TLR4, NLRP3 and CASP1 (Fig. 5H). Taken together, these results demonstrate that the C5a–C5aR1 axis regulates the TLR4 and NLRP3 signaling pathway in NASH.

A previous study reported that C5aR1 deficiency has an effect on macrophage phenotype in the kidney following renal ischemia/reperfusion insult [20]. Several lines of evidence have shown that the polarization of macrophage has an impact on the progression of NASH [25, 26]. Moreover, Orr et al. [27] reported that TLR4 deficiency promotes the alternative activation of adipose tissue macrophage. We therefore tested the function of C5aR1 in the phenotype changes of macrophages. Our results of western blot showed that C5aR1 loss decreased the expression of iNOS and CD86, but increased the expression of CD163 and CD206 (Fig. 5I). The results of qRT-PCR revealed that the hepatic level of iNOS was downregulated; in contrast, the expression level of CD163 was upregulated in C5aR1 deficient mice (Fig. 5J). These results demonstrate that C5aR1 deficiency promotes the differentiation of macrophage M2 phenotype in NASH mice.

KEGG pathway analysis showed that C5aR1 is also involved in mediating the signaling pathway of fatty acid metabolism (Fig. 5B). The fatty acid metabolism associated genes such Srebf1, Scd1 and Fasn was downregulated in C5aR1 deficient mice. Previous studies reported that AKT signaling pathway is involved in upregulating the expression of SREBP1c [28]. Several lines of evidence showed that C5a/C5aR1 signaling activates the AKT signaling pathways [29]. We speculated that C5aR1 participates in lipogenesis by regulating the AKT signaling pathway. Our result showed that C5aR1 deficiency reduced the ratio of p-AKT/AKT (Fig. 5K). As shown in Fig. 4F, the expression of Srebf1 and Fasn was decreased in the C5aR1 deficient mice. Taken together, C5aR1 deficiency prevents the progression of NASH by inhibiting the Toll-like receptor signaling pathway, promoting the differentiation of macrophage M2 phenotype and mediating the AKT signaling pathway.

### PMX-53 treatment prevents the progression of NASH in mice

**Fig. 6:**
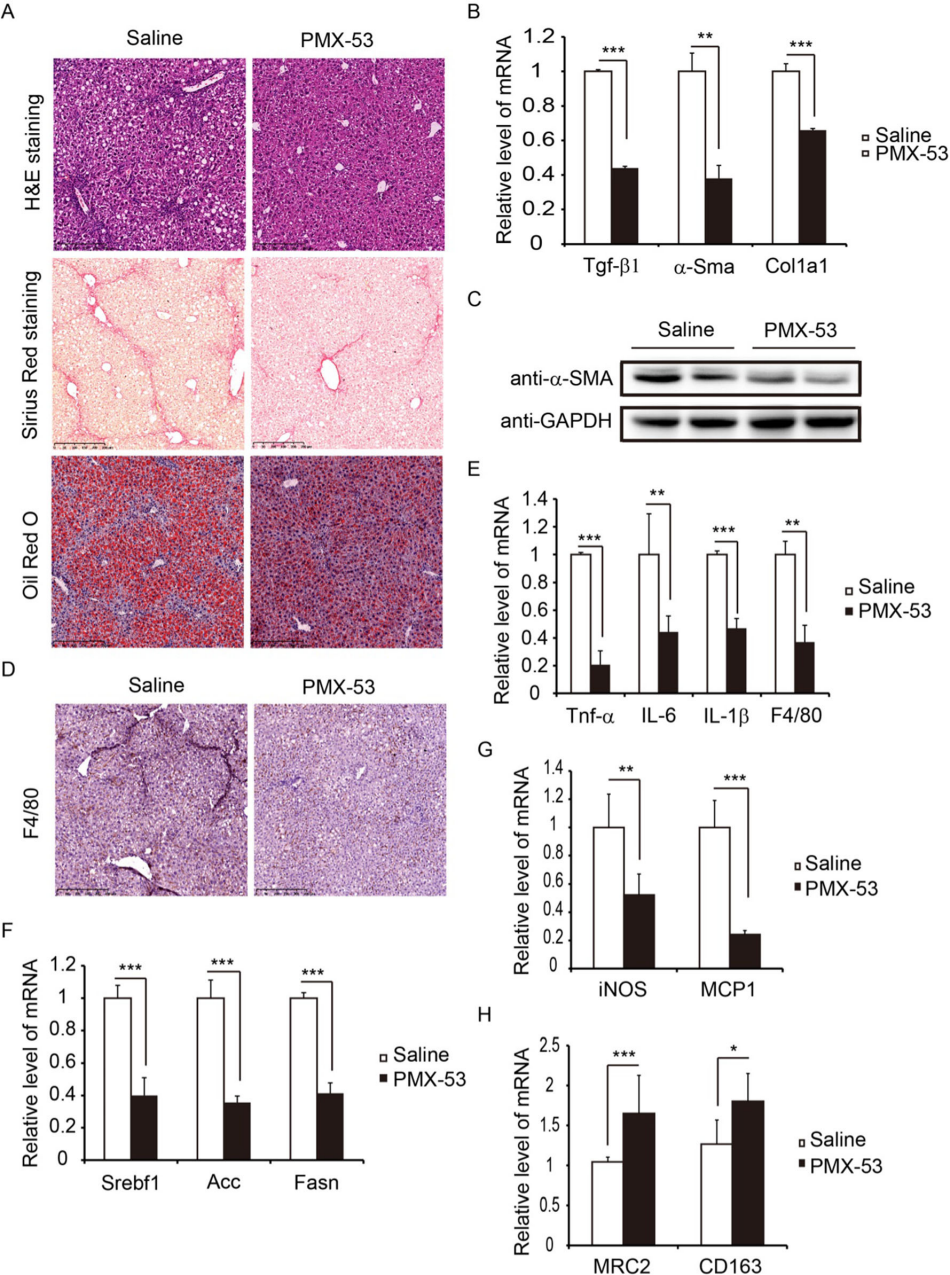
PMX-53 treatment suppresses the progression of NASH in mice. **A** Liver tissue sections were assessed by H&E staining (upper), Sirius Red staining (mid), and Oil Red O staining (lower). Scale bar: 250 μm. **B** The mRNA levels of Tgf-β1, α-Sma, and Col1a1. **C** The protein level of α-SMA was determined by western blot. **D** Immunohistochimical staining of F4/80. Scale bar: 250 μm. **E** The mRNA levels of Tnf-α, IL-6, IL-1β and F4/80. **F** The mRNA levels of Srebf1, Acc and Fasn were determined by qRT-PCR. **G** The mRNA levels of iNOS and MCP1. **H** The mRNA levels of MRC2 and CD163. These data of relative mRNA level are representative of three independent experiments. Results are presented as mean ± SD. *P < 0.05, **P < 0.01, ***P < 0.001.* n* = 6 per group

Our data demonstrate that C5aR1 is a potential target for the intervention of NASH. We then tested the therapeutic effect of the C5aR1 antagonist, PMX-53, in the NASH mice by treating over the last 4 weeks. H&E and Sirius Red staining showed reduced hepatic fibrosis in the PMX-53-treated mice (Fig. 6A). Furthermore, the result of qRT-PCR showed that the pro-fibrotic genes such as Tgf-β1, α-Sma and Col1a1 were greatly lowered by treatment of PMX-53 (Fig. 6B). Similarly, the protein level of α-SMA was decreased by PMX-53 treatment (Fig. 6C). Immunohistochemistry analysis showed that the deposition of F4/80 in liver was decreased in PMX-53-treated mice, and the mRNA level of F4/80 got the similar result (Fig. 6D and E). At the same time, the hepatic mRNA levels of inflammation associated genes such as Tnf-α, IL-6, and IL-1β were downregulated by treatment with PMX-53 (Fig. 6E). Oil red staining displayed that PMX‑53 treatment reduced lipid droplet accumulation in the liver (Fig. 6A). The hepatic expression levels of lipogenesis associated genes were decreased in the PMX-53 treatment group compared to control group (Fig. 6F). Furthermore, the results of qRT-PCR showed that PMX-53 treatment decreased the mRNA levels of macrophage M1 marker such as iNOS and MCP1, contrarily, upregulated the expression of M2 marker such as MRC2 and CD163 (Fig. 6G and H). Overall, blockade of C5aR1 resulted in a reduction of hepatic fibrosis, inflammatory response, and steatosis in NASH mice.

## Discussion

NASH, characterized by steatosis, inflammation and fibrosis, is an advanced form of NAFLD, which can lead to serious end-stage liver disease, such as hepatocellular carcinoma. Although some studies have reported that the C5a–C5aR1 axis exerts a crucial function in the development of inflammation and fibrosis in several diseases, the effects of the C5a–C5aR axis on the progression of NASH are still unclear. In this study, we examined the functions of the C5a–C5aR1 axis in the progression of chemically induced NASH in mice and explored the underlying mechanisms. We have been able to get some conclusions. Firstly, our results indicate that the C5a–C5aR1 axis is involved in mediating the development of hepatic fibrosis, inflammation and steatosis in a mouse model of NASH. C5 or C5aR1 deficiency diminished hepatic fibrosis, inflammatory response, and lipid accumulation in NASH. Mechanistically, C5aR1 deletion alleviates the progression of NASH by regulating Toll-like receptor signaling pathway and promoting the differentiation of macrophage M2 phenotype. Moreover, we found that administration of PMX-53, the C5aR1 antagonist, significantly reduced liver fibrosis, inflammation and steatosis in mice. Our data demonstrate that the C5a-C5aR1 axis may be regarded as a potential therapeutic target for hepatic fibrosis, inflammation and steatosis in NASH.

To investigate the effect of the C5a–C5aR1 axis on fibrosis, we used a WD and CCl_4_ treatment for 12 weeks to induce NASH model, because this model results in a rapid progression to severe NASH with fibrosis. Of note, this model showed a higher similarity to human NASH [22]. In this study, we found that complement system was activated in a CCl_4_-induced NASH model in mice. Our data demonstrated that C5 deficiency alleviated hepatic steatosis and inflammation in NASH mice. These results are supported by a previous study in murine nonalcoholic liver disease model [14]. Previous studies showed that C5a/C5aR1 signaling mediates the PI3K/Akt signaling pathways [29]. Yecies et al. [28] reported that AKT signaling pathway upregulates the expression of SREBP1c. In our study, the result of transcriptional profiling revealed that C5aR1 deficiency downregulated the expression of fatty acid metabolism associated genes such as Srebf1, Scd1 and Fasn. Our result confirmed that C5aR1 absence reduced the ratio of p-AKT/AKT and decreased the expression of Srebf1 and Fasn in the liver of NASH mice. Moreover, our results displayed that C5 deficiency decreases the level of oxidative stress. Oxidative stress is considered as “the second hit” and one of key factors to promote the progression of NASH [7]. In addition, we found that C5 deficiency resulted in a reduction of fibrosis in chemically induced NASH mice. C5 deficiency decreases the expression of TGF-β1 and α-SMA in NASH mice. Previous studies had shown that C5 is a quantitative trait gene associated with liver fibrosis in chronic hepatitis C virus infection [16, 30]. Another study demonstrated that C5 deficiency delays the progression of biliary fibrosis in bile duct-ligated mice [18]. A study by Sendler et al. [31] suggested that C5 mediates the development of fibrosis in chronic pancreatitis in mice. Xu et al. [17] reported that C5a activated HSCs and upregulated the expression of α-SMA and collagen, stimulating the progression of fibrosis in patients with chronic hepatitis B. These results further demonstrate that complement C5 is closely related to the progression of fibrosis. While, Seidel et al. [32]reported that anti-C5 antibody treatment did not reduce the development of NASH in Ldlr^−/−^.Leiden mice, which is different from our observations. This difference may be correlated to the NASH model used. In addition, anti-C5 treatment was only performed in the advanced stage of NASH.

C5a is identified as a chemotactic and inflammatory factor, which plays a pivotal role in inflammatory response by interacting with the receptor C5aR1. A study of Peng et al. [20] indicated that C5a–C5aR1 contributes to chronic post-ischemic fibrosis in a model of renal ischemia/reperfusion injury. We speculate that C5 deficiency attenuates liver fibrosis through the anaphylatoxin receptor C5aR1 in NASH mice. Our results indicated that C5aR1 deficiency diminishes the distribution of macrophage and further downregulates the expression of inflammation and pro-fibrotic associated genes. C5aR1 is widely expressed in a variety of cells, including macrophage and HSCs. Macrophages produce several pro-inflammatory factors, such as TNF-α, IL-6 and IL-1β, which contributes to the progression of inflammation and fibrosis. In addition, TNF-α is involved in regulating lipid metabolism [33]. Mechanistically, we unveiled that C5aR1 deletion delayed the progression of inflammation and fibrosis by regulating the Toll-like receptor signaling pathway and NOD-like receptor signaling pathway. A previous study showed that C5aR1 plays a critical role in the induction of liver fibrosis [34]. Gu et al. [35] reported that C5aR contributes to the pathogenesis of pulmonary fibrosis. Previous studies showed that Toll-like receptor signaling pathway is involved in the pathogenesis of NAFLD [36]. Several lines of evidence revealed that blockade of NLRP3 inflammasome activation alleviates liver inflammation and fibrosis in NAFLD [37–39]. The evidence demonstrated that loss of C5aR1 suppresses the NFκB pathway, which is involved in regulating the expression of inflammatory factors. Moreover, our study indicated that C5aR1 deletion promotes hepatic macrophage phenotype shift from M1 to M2. As we know, M2 macrophages are related to anti-inflammatory response and anti-fibrosis. Overall, our study demonstrates that C5aR1 deficiency has reduced effect on hepatic steatosis, inflammation and fibrosis in NASH mice.

To assess the clinical application of our research, we further explored the therapeutic effect of C5aR1 antagonist PMX-53 in NASH mice. We found that administration of PMX-53 also reduced hepatic steatosis, inflammation and fibrosis. Blockade of C5aR1 promoted the differentiation of macrophage M2 phenotype. As a result of our findings, we believe that C5aR1 may be a candidate target for drug development and therapy of NASH. In this study, we did not investigate the function of C5L2, which was considered as another C5a receptor. The roles of C5L2 in the progression of NASH need to be evaluated in the future work.

In summary, the present study demonstrates that the C5a-C5aR1 axis is strongly associated with the progression of NASH. C5aR1 deletion or blockade of C5aR1 with antagonist alleviates hepatic steatosis, inflammation, and fibrosis in NASH mice. Blockade of the C5a–C5aR1 axis may be an intervention strategy for the progression of NASH.

## Supplementary Information

Below is the link to the electronic supplementary material.

Supplementary file1 (DOCX 277 kb)
