# Supplementary material for: Blockade of C5aR1 alleviates liver inflammation and fibrosis in a mouse model of NASH by regulating TLR4 signaling and macrophage polarization
Source: J Gastroenterol. 2023 May 25;58(9):894–907. doi: 10.1007/s00535-023-02002-w (PMC10423130; doi:10.1007/s00535-023-02002-w)
Supplement: Supplementary file 1 — Supplementary file1 (DOCX 277 kb) [file 535_2023_2002_MOESM1_ESM.docx]

Supplementary data


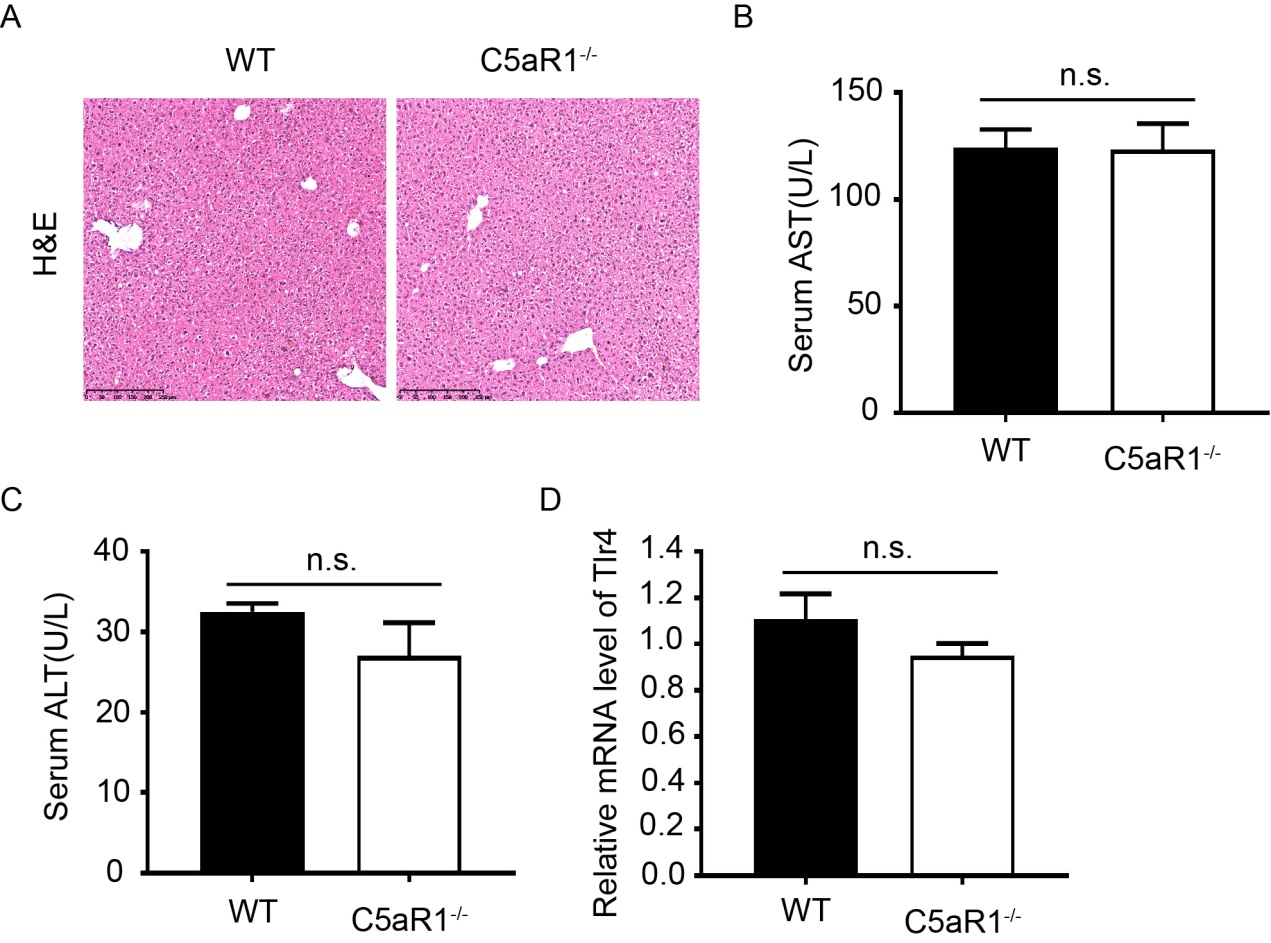


Supplementary Figure 1 C5aR1^-/-^ and wild type mice fed with normal diet.

A. H&E staining of liver tissue sections. B. Serum level of AST. C. Serum level of ALT. D. The mRNA level of Tlr4.

Supplementary Table 1 Primers used in this study

| **Gene** | **Forward primer** | **Reverse primer** |
| --- | --- | --- |
| IL-6 | TGATGCACTTGCAGAAAACA | ACCAGAGGAAATTTTCAATAGGC |
| Tnfα | CCACCACGCTCTTCTGTCTAC | AGGGTCTGGGCCATAGAACT |
| IL-1β | TGTGAAATGCCACCTTTTGA | GGTCAAAGGTTTGGAAGCAG |
| F4/80 | TCAAGGCCATTGCCCAGATT | CATCCCGTACCTGACGGTTG |
| αSma | CTACTGCCGAGCGTGAGATT | CGTTCTGGAGGGGCAATGAT |
| Col1a1 | GCACGTCTGGTTTGGAGAGA | TCCAAGGGAGCCACATCGAT |
| Tgfβ1 | AGTGGCTGAACCAAGGAGAC | CTGTGTGTCCAGGCTCCAAA |
| Fasn | GCCGTGTCCTTCTACCACAA | GGATCGGAGCATCTCTGGTG |
| Acc | TGAGGAGGACCGCATTTATC | GAAGCTTCCTTTGTGACCAG |
| Srebf1 | CGGGACAGCTTAGCCTCTAC | TCCATTGCTGGTACCGTGAG |
| iNOS | CGTTCCTGGAGGTGCTTGAA | TGGAAGCCACTGACACTTCG |
| MCP1 | CACTCCCGTCCTTACATGGC | GAGCTCCAAGGGTGACAGTG |
| MRC2 | GTGGGACGGTTCTGTCCATT | GCCTCCTTTGGGGTTGAAGT |
| CD163 | GAAGCGACGACAGATTCAGC | TGTGCCTCTGAATGACCCCT |
| Tlr4 | TTGCTGGGGCTCATTCACTC | AGACTCGGCACTTAGCACTG |
| Nlrp3 | CTTCTGCACCCGGACTGTAA | TAGCAGTGAAGAGCAGTGCG |
| GAPDH | CCCACTAACATCAAATGGGG | CCTTCCACAATGCCAAAGTT |
